# Supplementary material for: In vivo conformational space and defects of misfolded CFTR variants by covalent protein painting
Source: Nat Commun. 2025 Nov 19;16:10131. doi: 10.1038/s41467-025-63354-w (PMC12630588; doi:10.1038/s41467-025-63354-w)
Supplement: Supplementary file 1 — Supplementary Information [file 41467_2025_63354_MOESM1_ESM.pdf]

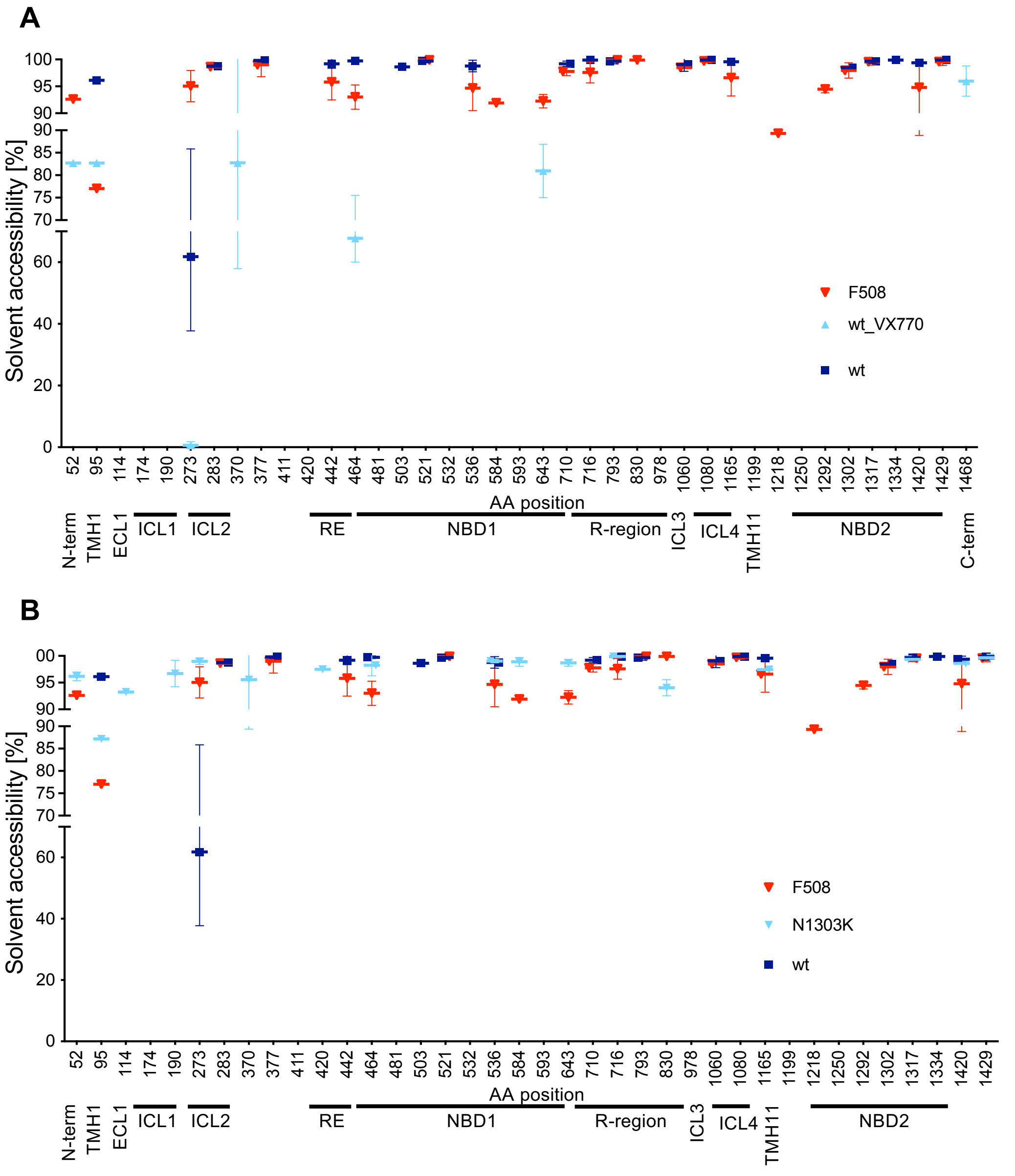

Supplementary Figure 1. A. Solvent accessibility of all quantified CFTR lysines upon activation with VX-770 (n=3). **B**. Solvent accessibility of all quantified N1303K CFTR lysines compared to ΔF508 and wt CFTR. N1303K (n=3).

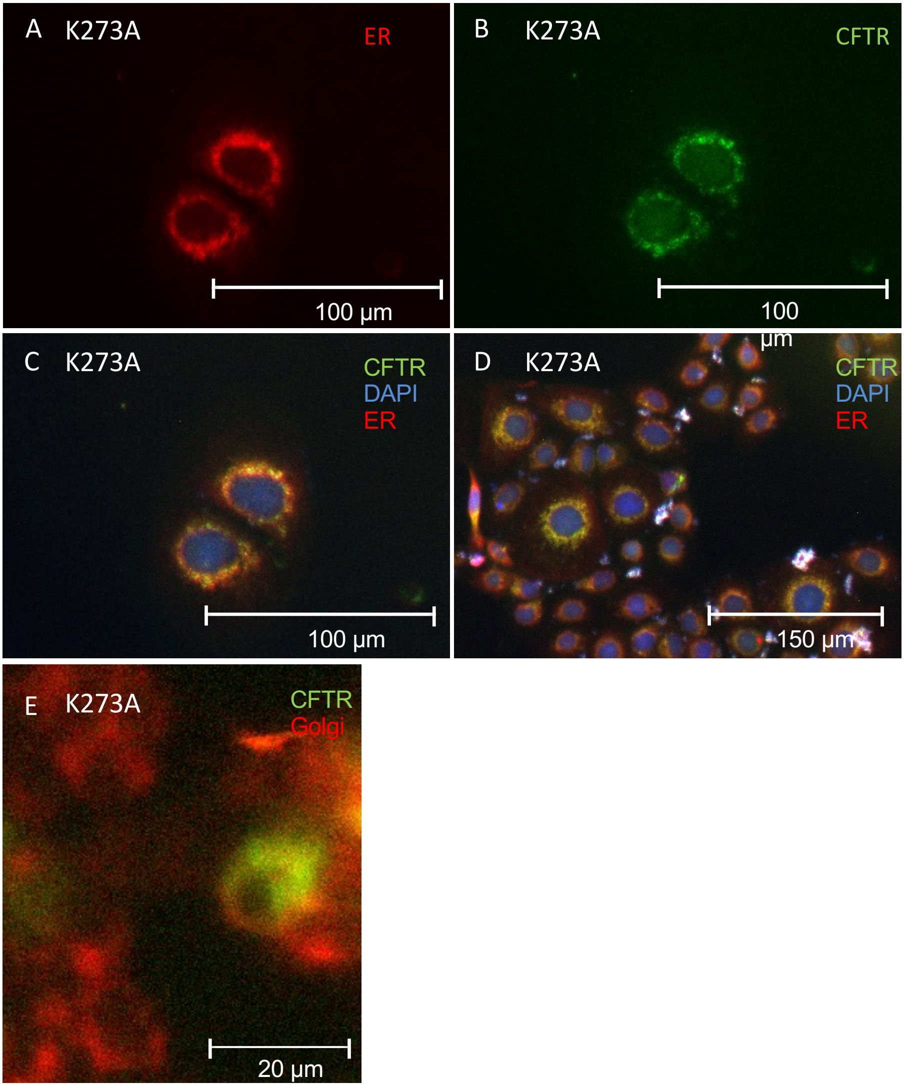

Supplementary Figure 2. ER and Golgi localization of the K273A CFTR mutant was probed in HEK293T cells transiently transfected with GFP-tagged K273A CFTR (green), either in fixed cells stained with the ER Cytopainter kit (red) (A-D) or in live cells using the Golgi Red detection probe (E). Nuclei were counterstained with DAPI in A-D. Images are representative of biological replicates (n=3)

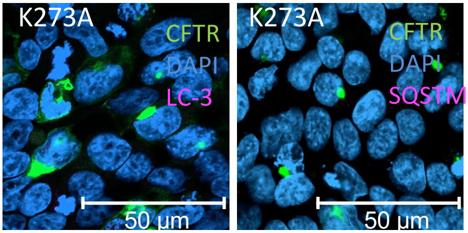

Supplementary Figure 3. HEK293T cells transiently transfected with GFP-tagged K273A CFTR were stained for autophagy markers SQSTM (pink) or cleaved LC-3 (pink).

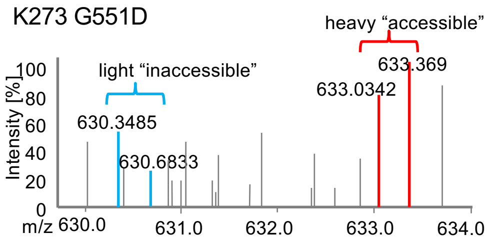

Supplementary Figure 4. Raw MS1 spectrum containing the precursor ions of the peptide containing K273 in G551D CFTR. Light and heavy precursors and measured m/z for each are indicated.

	wt (HBE41o-)	wtK273A (HEK)
K442	99.2 +/- 0.8	94.9 +/- 2.9
K464	99.7 +/- 0.2	97.4 +/- 1.9
K536	98.8 +/- 1.1	98.4 +/- 1.5
K716	99.9	98.5 +/-2.5
K793	99.7 +/- 0.5	98.5
K1420	99.4 +/- 0.6	97.8 +/-1.3

Supplementary Table 1. Comparison of solvent accessibility of residues in wt K273A CFTR expressing HEK cells and wt CFTR expressing HBE41o- cells.
