# Supplementary material for: In vivo conformational space and defects of misfolded CFTR variants by covalent protein painting
Source: Nat Commun. 2025 Nov 19;16:10131. doi: 10.1038/s41467-025-63354-w (PMC12630588; doi:10.1038/s41467-025-63354-w)
Supplement: Supplementary file 2 — Description of Additional Supplementary Files [file 41467_2025_63354_MOESM2_ESM.pdf]

Description of Additional Supplementary Files

Supplementary Data 1. Enriched GO-terms in K273A interactome with p-values and adjusted p-values.
