# Supplementary material for: In vivo conformational space and defects of misfolded CFTR variants by covalent protein painting
Source: Nat Commun. 2025 Nov 19;16:10131. doi: 10.1038/s41467-025-63354-w (PMC12630588; doi:10.1038/s41467-025-63354-w)
Supplement: Supplementary file 4 — Reporting Summary [file 41467_2025_63354_MOESM4_ESM.pdf]

nature portfolio

Corresponding author(s): Yates JR; Pankow S.

Last updated by author(s): 05/27/2025

Reporting Summary

Nature Portfolio wishes to improve the reproducibility of the work that we publish. This form provides structure for consistency and transparency in reporting. For further information on Nature Portfolio policies, see our Editorial Policies and the Editorial Policy Checklist.

Statistics

For all statistical analyses, confirm that the following items are present in the figure legend, table legend, main text, or Methods section.

n/a	Confirmed	
☐	☒	The exact sample size (*n*) for each experimental group/condition, given as a discrete number and unit of measurement
☐	☒	A statement on whether measurements were taken from distinct samples or whether the same sample was measured repeatedly
☐	☒	The statistical test(s) used AND whether they are one- or two-sided<br>*Only common tests should be described solely by name; describe more complex techniques in the Methods section.*
☐	☒	A description of all covariates tested
☐	☒	A description of any assumptions or corrections, such as tests of normality and adjustment for multiple comparisons
☐	☒	A full description of the statistical parameters including central tendency (e.g. means) or other basic estimates (e.g. regression coefficient) AND variation (e.g. standard deviation) or associated estimates of uncertainty (e.g. confidence intervals)
☐	☒	For null hypothesis testing, the test statistic (e.g. *F*, *t*, *r*) with confidence intervals, effect sizes, degrees of freedom and *P* value noted<br>*Give P values as exact values whenever suitable.*
☒	☐	For Bayesian analysis, information on the choice of priors and Markov chain Monte Carlo settings
☒	☐	For hierarchical and complex designs, identification of the appropriate level for tests and full reporting of outcomes
☒	☐	Estimates of effect sizes (e.g. Cohen's *d*, Pearson's *r*), indicating how they were calculated

Our web collection on statistics for biologists contains articles on many of the points above.

Software and code

Policy information about availability of computer code

Data collection	Mass spectra were collected with the respective native Thermo Xcalibur version on the instrument (Thermo) and using settings described in detail in the Methods section. Mass spectra collected on the TimsTofPro were acquired with Bruker Hystar version 6.2 or earlier.
Data analysis	For analysis of mass spectrometry data, raw-files were extracted with RawConverter and MS/MS spectra were searched with ProLuCID against the human Uniprot database (release Nov 2019) and filtered with DTASelect using the Integrated Proteomics Pipeline (IP2, Integrated Proteomics Applications), version 6.1.2. Census (IP2) was used to quantify MS1 areas for each peptide and ProteinClusterQuant (PCQ, https://github.com/proteomicsyates/ProteinClusterQuant) to analyze ratio values for each lysine site.

For manuscripts utilizing custom algorithms or software that are central to the research but not yet described in published literature, software must be made available to editors and reviewers. We strongly encourage code deposition in a community repository (e.g. GitHub). See the Nature Portfolio guidelines for submitting code & software for further information.

Data

Policy information about availability of data

All manuscripts must include a data availability statement. This statement should provide the following information, where applicable:

- Accession codes, unique identifiers, or web links for publicly available datasets
- A description of any restrictions on data availability
- For clinical datasets or third party data, please ensure that the statement adheres to our policy

Datasets are deposited at the MassIVE repository under accession number MSV000093050 and will be made public upon publication.

Research involving human participants, their data, or biological material

Policy information about studies with human participants or human data. See also policy information about sex, gender (identity/presentation), and sexual orientation and race, ethnicity and racism.

Reporting on sex and gender	No human participants were involved in the study nor were human data used.
Reporting on race, ethnicity, or other socially relevant groupings	No human participants were involved in the study nor were human data used.
Population characteristics	No human participants were involved in the study nor were human data used.
Recruitment	No human participants were involved in the study nor were human data used.
Ethics oversight	No human participants were involved in the study nor were human data used.

Note that full information on the approval of the study protocol must also be provided in the manuscript.

Field-specific reporting

Please select the one below that is the best fit for your research. If you are not sure, read the appropriate sections before making your selection.

☒ Life sciences ☐ Behavioural & social sciences ☐ Ecological, evolutionary & environmental sciences

For a reference copy of the document with all sections, see nature.com/documents/nr-reporting-summary-flat.pdf

Life sciences study design

All studies must disclose on these points even when the disclosure is negative.

Sample size	This study used different cell lines. For every experiment at least three biological replicates were collected and measured, which is common practice in the MS field and analytical sciences as effect sizes are expected to be relatively consistent using cell line material. Results were cross-validated with different cell lines and by swapping the isotope encoding of the two different labels.
Data exclusions	No data were excluded.
Replication	Biological replicates were collected and measured at different dates and times and even measured on different instruments to minimize any potential batch effects. Replication attempts were successful across different replicates. The reported effects could also be reproduced in different cell lines as well as in "labelswap" experiments.
Randomization	No experimental groups were defined in this study. Samples were measured in random order.
Blinding	Blinding was not relevant to this study as no experimental groups were defined.

Reporting for specific materials, systems and methods

We require information from authors about some types of materials, experimental systems and methods used in many studies. Here, indicate whether each material, system or method listed is relevant to your study. If you are not sure if a list item applies to your research, read the appropriate section before selecting a response.

Materials & experimental systems

n/a	Involved in the study
☐	☒ Antibodies
☐	☒ Eukaryotic cell lines
☒	☐ Palaeontology and archaeology
☒	☐ Animals and other organisms
☒	☐ Clinical data
☒	☐ Dual use research of concern
☒	☐ Plants

Methods

n/a	Involved in the study
☒	☐ ChIP-seq
☒	☐ Flow cytometry
☒	☐ MRI-based neuroimaging

Antibodies

Antibodies used

CFTR was detected using mouse monoclonal antibodies 24.1 (ATCC) and M3A7 (EMD Millipore). Antibodies against b-actin (AC-15, Sigma) and Na+/K+ATPase a (H300, sc28800, Santa Cruz) were used for normalization. Antibodies against LC3A/B (Cell Signaling Technologies, cat no. 12741S) and SQSTM1 (Cell Signaling Technologies, cat no. 8025S) were used to detect autophagy.

Validation

The M3A7 anti-CFTR antibody used in this study was acquired from Millipore with manufacturers quality certifications. A new batch of antibody was tested with positive and negative controls. Controls include exogenous CFTR overexpression (positive control) and cell lines that do not express CFTR (negative control). The 24.1 anti-CFTR antibody was purchased from ATCC as hybridoma cell line and the antibody was produced at the TSRI antibody core. The antibody batches used for this study have been rigorously tested with positive and negative controls using exogenous CFTR overexpression and cell lines that do not express CFTR.
Both of these antibodies have been used in the laboratory for over a decade and have been cited in thousands of publications. b-actin (AC-15, Sigma) is a standard antibody validated by many different methods such as IPs and identification of the target, western blotting, knockdowns and knockout models and published in >10,000 publications. The Na+/K+ATPase a (H300, sc28800, Santa Cruz) antibody has been cited in >95 publications (https://www.scbt.com/p/na-plus-k-plus-atpase-alpha-antibody-h-300), has been used as a standard in the CF field. The SQSTM1 antibody was validated by western blot using knockout cell lines and has been cited in over 300 publications according to the manufacturer. The LC3A/B antibody has been cited in >1000 publications and has validation data as autophagy marker on the manufacturer website.

Eukaryotic cell lines

Policy information about cell lines and Sex and Gender in Research

Cell line source(s)

Cell lines were obtained from Dr. Eric Sorscher's laboratory at University of Alabama, have been published and were confirmed to express the wt, ΔF508, N1303K or G551D CFTR by detecting CFTR and the relevant mutation using Mass Spectrometry.

Authentication

Cell lines were authenticated by confirming the expression of the wt, ΔF508, N1303K or G551D CFTR by detecting CFTR and the relevant mutation using Mass Spectrometry. Additionally, proteome analysis confirmed the bronchial origin of the cell line by detecting bronchial marker proteins.

Mycoplasma contamination

All cell lines tested negative for mycoplasma contamination.

Commonly misidentified lines
(See ICLAC register)

none

Plants

Seed stocks

The study does not involve the use of plant material

Novel plant genotypes

Describe the methods by which all novel plant genotypes were produced. This includes those generated by transgenic approaches, gene editing, chemical/radiation-based mutagenesis and hybridization. For transgenic lines, describe the transformation method, the number of independent lines analyzed and the generation upon which experiments were performed. For gene-edited lines, describe the editor used, the endogenous sequence targeted for editing, the targeting guide RNA sequence (if applicable) and how the editor was applied.

Authentication

Describe any authentication procedures for each seed stock used or novel genotype generated. Describe any experiments used to assess the effect of a mutation and, where applicable, how potential secondary effects (e.g. second site T-DNA insertions, mosiacism, off-target gene editing) were examined.
